# Internal medicine trainees' knowledge and confidence in using the American Society of Hematology Choosing Wisely guidelines in hemostasis, thrombosis, and non-malignant hematology

**DOI:** 10.1371/journal.pone.0197414

**Published:** 2018-05-16

**Authors:** Ariela L. Marshall, Sarah Jenkins, Amy S. Oxentenko, Alfred I. Lee, Mark D. Siegel, Joel T. Katz, Jatin M. Vyas, John Del Valle, Joseph R. Mikhael

**Affiliations:** 1 Division of Hematology, Department of Internal Medicine, Mayo Clinic, Rochester, Minnesota, United States of America; 2 Department of Laboratory Medicine and Pathology, Mayo Clinic, Rochester, Minnesota, United States of America; 3 Division of Biomedical Statistics and Informatics, Mayo Clinic, Rochester, Minnesota, United States of America; 4 Department of Internal Medicine, Mayo Clinic, Rochester, Minnesota, United States of America; 5 Division of Hematology, Yale School of Medicine, New Haven, CT, United States of America; 6 Department of Internal Medicine, Brigham and Women’s Hospital, Boston, MA, United States of America; 7 Department of Internal Medicine, Massachusetts General Hospital, Boston, MA, United States of America; 8 Department of Internal Medicine, University of Michigan, Ann Arbor, MI, United States of America; 9 Division of Hematology and Medical Oncology, Department of Medicine, Mayo Clinic, Phoenix, Arizona, United States of America; Charles P. Darby Children's Research Institute, UNITED STATES

## Abstract

**Background:**

Several specialty societies participate in the Choosing Wisely (CW) campaign in an attempt to reduce waste in health care spending. We surveyed internal medicine (IM) residents with an objective of classifying knowledge of and confidence in using the American Society of Hematology (ASH) CW principles in hemostasis, thrombosis, and non-malignant hematology.

**Methods:**

Multi-institutional study of IM residents at 5 academic training programs in the United States. A 10-question, case-based multiple choice test, with each question accompanied by a 5-point Likert-scale confidence assessment, was distributed electronically. Responses were summarized with frequencies and percentages or medians and ranges, as appropriate. Two sample t-tests or Wilcoxon rank-sum tests were used to compare confidence and knowledge scores.

**Results:**

Of 892 IM residents, 174 (19.5%) responded to all questions. Overall, residents answered a median of 7 of 10 questions correctly (range 2–10) and median resident confidence in their responses was 3.1 (on a 5-point scale). Correct responses were significantly associated with higher confidence for all but one question. Having a hematology rotation experience was significantly associated with more correct responses and with higher confidence (*p = 0*.*001* and *p<0*.*001*, respectively).

**Conclusions:**

IM residents at several academic hospitals have variable knowledge of ASH-CW guidelines in thrombosis and hemostasis/non-malignant hematology. Residents who have done hematology rotations, particularly a hematology consult rotation, were more likely to answer questions correctly and to be more confident that their answers were correct. Adequate clinical exposure and training in cost-effective care is essential to train clinicians who are cost-conscious in any specialty.

## Introduction

The American Board of Internal Medicine developed the *Choosing Wisely* (CW) campaign in an attempt to reduce waste in health care spending.[1] The American Society of Hematology (ASH) has actively engaged with the CW campaign and developed lists of hematology-related tests and treatments to avoid. ASH has also incorporated hematology-applicable recommendations from other specialty societies.[2–3] The majority of the ASH-CW recommendations (~80%) are focused on non-malignant hematologic conditions and disorders of thrombosis and hemostasis (venous thromboembolism, transfusion goals, ITP, etc). The ASH-CW guidelines have been in circulation for several years, but it is unclear how many physicians–including both hematologists and non-hematologists caring for patients with hematologic issues–are aware of these guidelines and incorporate them into clinical practice.

Trainees at academic medical centers are often exposed to guidelines and quality improvement initiatives during the course of their training. As such we were interested in exploring trainees’ familiarity and confidence incorporating the ASH-CW guidelines into their medical decision-making. Because these hematology guidelines are most directly applicable to trainees and practitioners in internal medicine (IM) and IM subspecialties, we wanted to explore this question from the standpoint of IM resident trainees.

## Methods

### Survey design

Ten multiple choice questions, each based on a single ASH-CW guideline in non-malignant hematology and/or thrombosis/hemostasis, were developed. The questions were reviewed by two faculty members (one the chair of the hematology division’s non-malignant hematology disease-oriented group and one the chair of the coagulation medicine disease-oriented group) as well as two IM trainees. Feedback was incorporated into the final version of the questions in S1 File. Answer explanations are also provided in S1 File. For each question, a corresponding confidence score (how confident the resident was that their response to the question was correct) was added, assessed using a Likert-type scale ranging from 1 (very low confidence) to 5 (very high confidence).

### Survey administration

This study was reviewed by the IRB at Mayo Clinic Rochester and deemed to fall into the "not research" category due to the nature of the study (IRB review number 16–008378). After approval by the Internal Medicine Research in Education (IM-REG) group at the first author’s institution, the survey was distributed in electronic format to IM residency directors at five academic IM programs nationwide. The residency directors subsequently distributed the survey to their trainees. All IM residency trainees [regardless of PGY status, resident type (preliminary, categorical, medicine-pediatrics, etc)] were eligible to participate. The survey remained open for one month, and a reminder email was sent to residents after two weeks. Answers were collected and the primary response data is available in S2 File with a coding description in S3 File.

### Statistical analysis

Nominal variables were summarized with frequencies and percentages. Continuous variables were summarized with means and standard deviations (SD), and medians and ranges, as appropriate. For each respondent, an overall percentage correct among the 10 knowledge items was calculated, and an overall confidence score was calculated as the average across the 10 confidence items. Two sample t-tests or ANOVA F-tests were used to compare confidence and knowledge scores between groups (i.e., comparison of average confidence between those who answered an item corrects vs incorrectly; comparison of average knowledge score between the three program years). Chi-square or Fisher’s exact tests were used to compare nominal variables between selected groups (i.e., association of high/very high confidence versus correct/incorrect answer for an item). Spearman rank-based correlations were calculated to measure the degree of association between knowledge and confidence scores. P-values less than 0.05 were considered statistically significant. All analyses were performed using SAS version 9.4 (SAS Institute Inc., Cary, NC).

## Results

### Resident characteristics and background

Of 892 IM residents, 201 (22.5%) responded to at least one question, and 174 (19.5%) responded to all knowledge questions and were termed “complete responders.” Analysis was restricted to the 174 complete responders and results are shown in **Table 1**. Of the 174 complete responders, 96 (56.1%) were male and the median age was 28.7 years (range 24.5–40.4). There were 151 (86.8%) were categorical IM residents, 21 (12.1%) preliminary residents, and 2 (1.1%) who classified themselves as “other.” 79 (45.4%) were at the PGY1 level, 41 (23.6%) at the PGY2 level, 51 (29.3%) at the PGY3 level and 3 (1.7%) who were PGY4 level or higher. 56 (32.2%) of residents had done at least one hematology rotation in residency by the time they completed the survey, most often inpatient malignant hematology (54, 31.0%) and hematology consult (33, 19.0%). Additionally, 28 (16.1%) had done a hematology elective during medical school. 53 (30.5%) were aware of the ASH-CW guidelines prior to participation in this study. In a subgroup analysis by residency site, there were significant differences between sites with respect to the percentage of residents who reported doing a hematology rotation during residency and who reported being aware of the ASH-CW guidelines.

**Table 1:** Demographics of complete responders.

Sex	N (%)
Male	96 (56.1%)
Female	75 (43.9%)
Missing	3
**Age (median, range)**	28.7 (24.5–40.4)
**Resident Type**	
Prelim	21 (12.1%)
Categorical	151 (86.8%)
Other	2 (1.1%)
**Year in Residency**	
PGY1	79 (45.4%)
PGY2	41 (23.6%)
PGY3	51 (29.3%)
Other	3 (1.7%)
**Residency Site**	
Site 1	40 (23.0%)
Site 2	21 (12.1%)
Site 3	69 (39.7%)
Site 4	18 (10.3%)
Site 5	26 (14.9%)
**Hematology Rotation during Residency?**	
Yes	56 (32.2%)
No	118 (67.8%)
**Type of Hematology Rotation?**	
Hematology Consult	33 (19.0%)
Inpatient malignant hematology	54 (31.0%)
Outpatient malignant hematology	6 (3.4%)
Outpatient non-malignant hematology	16 (9.2%)
Other	2 (1.1%)
**Hematology Elective during Medical School?**	
Yes	28 (16.1%)
No	146 (83.9%)
**Aware of ASH Choosing Wisely Guidelines?**	
Yes	53 (30.5%)
No	121 (69.5%)

### Responses to questions

**Table 2** depicts resident responses to each of the 10 questions, and the number of residents reporting either high (score = 4) or very high (score = 5) confidence that their response was correct. The percentage of residents answering questions correctly ranged from 44.3% (workup of heparin-induced thrombocytopenia) to 96.6% (avoidance of thrombophilia workup in the setting of provoked deep venous thrombosis). For 9 of 10 questions, residents who answered the question correctly were significantly more likely to report high or very high confidence that their answer was correct (*p≤0*.*05)*, and there was a trend towards this association in the tenth question as well (p = 0.0599). Overall, residents answered a median of 7 questions correctly (range 2–10) and the median confidence score was 3.1 (range 1.5–5.0).

**Table 2:** Test results (Complete Responders).

	N (%)	p-value*
**Question 1: Red Cell Transfusion Threshold**		0.006
Correct Response	161 (92.5%)	
High/Very High Confidence	77 (44.3%)	
**Question 2: Provoked DVT Workup**		0.0009
Correct Response	168 (96.6%)	
High/Very High Confidence	108 (62.1%)	
**Question 3: Elevated INR on Warfarin**		<0.0001
Correct Response	136 (78.2%)	
High/Very High Confidence	53 (30.5%)	
**Question 4: Provoked DVT Treatment**		0.06
Correct Response	130 (74.7%)	
High/Very High Confidence	81 (46.6%)	
**Question 5: Sickle Cell Anemia**		0.006
Correct Response	80 (46.0%)	
High/Very High Confidence	30 (17.2%)	
**Question 6: HIT Workup**		<0.0001
Correct Response	77 (44.3%)	
High/Very High Confidence	58 (33.3%)	
**Question 7: ITP Management**		<0.0001
Correct Response	132 (75.9%)	
High/Very High Confidence	34 (19.5%)	
**Question 8: Iron Deficiency Anemia**		0.003
Correct Response	108 (62.1%)	
High/Very High Confidence	55 (31.6%)	
**Question 9: Routine Bloodwork**		<0.0001
Correct Response	125 (71.8%)	
High/Very High Confidence	58 (33.3%)	
**Question 10: Pulmonary Embolism Workup**		0.02
Correct Response	107 (61.5%)	
High/Very High Confidence	73 (42.0%)	
**Overall**		
Median % correct (IQR)	70 (60–80)	
Range	20–100	

*T-test comparing average confidence level between correct versus incorrect responders

As residents progressed from PGY1 to PGY2 to PGY3, the average percent of answers correct increased from 64.2% to 72.9% to 77.1%, respectively (*p<0*.*001)*. Similarly, average confidence increased from 2.7 to 3.2 to 3.5 (*p<0*.*001)*. However, although there was a slight positive correlation between overall percent of answers correct and overall confidence, this association was not very strong (Spearman correlation 0.37), and this was was similar within each year in training (Spearman correlation 0.41, 0.35, and 0.34 for PGY1, PGY2, and PGY3, respectively).

**Table 3** shows differences in resident response based on participation in a hematology rotation during residency. Residents who had done hematology rotations were significantly more likely to answer more questions correctly (*p = 0*.*001)* and to report higher confidence that their answers correct (*p<0*.*001)* than residents who had not done any hematology rotations during residency. This appeared to be driven primarily by participation in a hematology consult rotation, as the association between rotation participation, correct responses, and confidence was not significant based on participation in inpatient or outpatient malignant hematology rotations. There was a trend towards higher confidence in residents who had participated in outpatient non-malignant hematology rotations (*p = 0*.*02)* though only 16 residents (9.2%) had done this rotation.

**Table 3:** Results based on hematology rotation experience.

	Yes	No	p-value
**Any hematology rotation**			
Mean % correct (SD)	76.1 (15.1)	67.6 (16.4)	0.001
Median correct (range)	80 (50–100)	70 (20–100)	
Mean confidence (SD)	3.5 (0.6)	2.9 (0.6)	<0.001
Median confidence (range)	4 (2–5)	3 (2–4)	
**Among those who did a hematology rotation (N = 56):**			
**Hematology Consult**			
Mean % correct (SD)	79.7 (15.3)	70.9 (13.5)	0.03
Median correct (range)	80 (50–100)	70 (50–90)	
Mean confidence (SD)	3.7 (0.6)	3.2 (0.7)	0.005
Median confidence (range)	4 (2–5)	3 (2–5)	
**Inpatient Malignant Hematology**			
Mean % correct (SD)	76.1 (15.3)	75 (7.1)	0.91
Median correct (range)	80 (50–100)	75 (70–80)	
Mean confidence (SD)	3.5 (0.6)	3.7 (0.8)	0.70
Median confidence (range)	4 (2–5)	4 (3–4)	
**Outpatient Malignant Hematology**			
Mean % correct (SD)	76.7 (13.7)	76 (15.4)	0.92
Median correct (range)	75 (60–100)	80 (50–100)	
Mean confidence (SD)	3.9 (0.7)	3.4 (0.6)	0.13
Median confidence (range)	4 (3–5)	3 (2–5)	
**Outpatient Non-Malignant Hematology**			
Mean % correct (SD)	79.4 (13.4)	74.8 (15.7)	0.30
Median correct (range)	80 (50–100)	80 (50–100)	
Mean confidence (SD)	3.8 (0.6)	3.4 (0.6)	0.02
Median confidence (range)	4 (3–5)	3 (2–4)	
**Other**			
Mean % correct (SD)	76.7 (13.7)	76 (15.4)	0.92
Median correct (range)	75 (60–100)	80 (50–100)	
Mean confidence (SD)	3.9 (0.7)	3.4 (0.6)	0.13
Median confidence (range)	4 (3–5)	3 (2–5)	

## Discussion

It is estimated that up to 30% of all medical spending is unnecessary and does not add value, and that physician decision-making is estimated to account for about 80% of health care expenditures.[4] Given this, campaigns such as Choosing Wisely which aim to reduce unnecessary spending and promote high-value, cost-effective care are of paramount importance in todays’ health care systems. The Choosing Wisely campaign started in the United States in 2012 and has spread to several other countries, with the aim of changing the culture of medical care from one of overuse to one of evidence-based, patient-centered, cost-effective testing and treatment.[5–6] Studies indicate that adherence to Choosing Wisely recommendations varies widely, often impacted by individual practice site characteristics.[7] Nonadherence has been associated with significantly increased costs of care.[8] Initiatives to increase adherence to CW guidelines have been demonstrated to improve practices and reduce costs at academic medical centers.[9]

Initial exposure to the importance of high-value, cost-effective care would ideally occur at the medical trainee level, particularly as there is evidence that residents trained in hospitals with high health care expenditure patterns are more likely to provide subsequently provide more costly care as practicing providers.[10] Initiatives to increase medical trainees’ exposure to cost-conscious care are becoming more common. Cost-effective, high-quality care is a component of the Accreditation Council for Graduate Medical Education (ACGME) Clinical Learning Environment Review (CLER) “pathways to excellence” and while relatively few internal medicine programs have a formal curriculum in cost-conscious care, almost 50% reported that they are working to create such programs.[11–12] A survey of emergency medicine residents found that almost all supported the Choosing Wisely campaign, though many barriers to implementation of the recommendations were cited[13]

In this study, we surveyed IM residents at several large academic medical centers regarding both knowledge of and confidence with the ASH-CW guidelines in non-malignant hematology and thrombosis/hemostasis. Clinical experience, particularly on a hematology consult rotation, was associated with both increased knowledge (ability to answer content-based questions correctly) and increased confidence that answers were correct. For almost all questions, there was a significant correlation between knowledge and confidence (residents who answered questions correctly were more confident that their answers were correct). We believe that these results demonstrate both the importance of adequate exposure to hematology during IM residency training as well as the larger-scale importance of educational programs designed to familiarize residents with the principles of high-value care.

Our study is one of the first to explore resident trainees’ knowledge of and confidence in Choosing Wisely guidelines, and one of the studies’ major strengths is the multi-institutional nature. In assessing specialty-specific (hematology) knowledge using questions tailored to be appropriate for the resident physician level, we were able to assess knowledge of basic principles of cost-effective care in non-malignant hematology/thrombosis and hemostasis which should be mastered by all graduates of internal medicine training programs regardless of post-residency specialty choice. Therefore, this study is widely generalizable to all other academic medicine residency programs and in addition provides a platform for similar studies in other specialty areas that all residents should be aware of as they graduate to independent practice. Additionally, similar studies could be developed for residents in other specialty areas (pediatrics, obstetrics-gynecology, radiology, etc) to assess knowledge of their specialty societies’ Choosing Wisely guidelines prior to graduation from residency.

It is somewhat concerning that less than one-third of respondents were aware of the ASH-CW guidelines prior to the survey. We highly encourage initiatives to increase awareness of these guidelines at the medical student and resident level, prior to graduation to independent practice. We also note that residents performed most poorly on questions regarding sickle cell anemia and heparin-induced thrombocytopenia, both of which are common conditions encountered by both hematologists and physicians of many other specialties and are associated with significant morbidity, mortality, and high cost of care.[14–15] Educational and protocol-driven interventions to increase awareness and appropriate management of these conditions, such as the American Society of Hematology’s Sickle Cell Disease Initiative (the first time ASH has developed an initiative in support of a single disease) seem highly necessary.[16]

One weakness of this study is the relatively low response rate, which could also reflect bias on the part of which residents chose to complete the survey (for example, respondents may have a better knowledge base and may have performed better on the questions than non-respondents). However, with a relatively large total number of participants (almost 200) and with the relatively broad distribution of respondents in terms of sex, year of training, and training site, we believe that our results are still representative of resident knowledge/confidence on average for residents in a large academic training program. Because smaller community-based programs were not included in the survey, the results may not be as widely generalizable this type of training programs. Another weakness is the possible confounding bias between year in residency and the benefit gained by participation in a hematology consult rotation. We found that resident knowledge and confidence increased both with year in residency and also with participation in a hematology consult rotation. Later-year residents were more likely to participate in a consult rotation (*p-value* for trend *<0*.*0001*). Therefore, it is possible that the overall experience gained with progression in residency, rather than the experience from the hematology consult rotation specifically was the driver of the increased knowledge and confidence.,. However, participation in other types of hematology rotations was also significantly correlated with year of residency, and aswe did not see similar increases in knowledge and confidence based on participation in other types of hematology rotations, it seems more likely that the consult-specific experience was the major driver of increased knowledge/confidence rather than year in residency alone.

We hope that this study demonstrates the importance of cost-conscious training in subspecialty areas for medical trainees and provides a framework for future educational curricular development. Curricula in high-value care and decision support have been well-received by medical trainees, and housestaff-led initiatives have led to significant reductions in unnecessary spending.[17–19] Crowdsourcing was used to solicit ideas from both leaders in medical education as well as trainees themselves as part of the “Choosing Wisely Challenge.”[20] Additional initiatives could be adapted to develop curricula and promote educational rotations in subspecialty areas. The ultimate goal is to create training programs that teach and role-model high-value, cost-conscious care in both general and subspecialty areas so that trainees graduates develop “good habits” early on. This will ideally lead to a workforce equipped with the knowledge and confidence that they need to provide high-value, cost-conscious care as practicing physicians and improve the overall quality and value of our health care system as a whole.

## Supporting information

S1 File Questions and answers included in the survey. (DOCX)

S2 File Database of resident responses (Primary Data). (XLSX)

S3 File Codebook used to code the responses in numeric format for analysis. (DOCX)
