# Supplementary material for: Internal medicine trainees' knowledge and confidence in using the American Society of Hematology Choosing Wisely guidelines in hemostasis, thrombosis, and non-malignant hematology
Source: PLoS One. 2018 May 16;13(5):e0197414. doi: 10.1371/journal.pone.0197414 (PMC5955511; doi:10.1371/journal.pone.0197414)
Supplement: S1 File — (DOCX) [file pone.0197414.s001.docx]

**QUESTIONS AND ANSWERS**

1A. A 65-year old patient with a history of leukemia who recently received chemotherapy, and no history of cardiac disease, presents with a hemoglobin of 6.9. He is admitted to the hospital for management. What is your recommended course of action?

1. Transfuse 2 units of packed red cells
2. Transfuse 1 unit of packed red cells
3. Start erythropoietin stimulating agent (ESA) such as Epogen
4. Give an infusion of intravenous iron

1B. On a scale of 1-5, with 1 being very low and 5 being very high, what is your level of confidence that your answer to the previous question is in accordance with ASH Choosing Wisely recommendations?

1 2 3 4 5

*Answer: B*

*Based on the recommendation “Don’t transfuse more than the minimum number of red blood cell (RBC) units necessary to relieve symptoms of anemia or to return a patient to a safe hemoglobin range (7 to 8 g/dL in stable, non-cardiac in-patients).”*

2A. Two days after being hospitalized for a motor vehicle accident, a 45-year old gentleman develops lower extremity swelling and is diagnosed with a deep venous thrombosis. He does not know of any family history of major venous thromboembolism. He is started on heparin (as bridging agent) and warfarin therapy. What is the most appropriate course of action in terms of further workup?

1. Send factor V Leiden and prothrombin gene mutation testing only
2. Send protein C and S and antithrombin testing only
3. Send a complete thrombophilia workup (Factor V Leiden, prothrombin gene mutation, protein C and S levels, antithrombin level, and antiphospholipid antibody testing)
4. Do not send any thrombophilia testing

2B. On a scale of 1-5, with 1 being very low and 5 being very high, what is your level of confidence that your answer to the previous question is in accordance with ASH Choosing Wisely recommendations?

1 2 3 4 5

*Answer: D*

*Based on the recommendation “Don’t test for thrombophilia in adult patients with venous thromboembolism (VTE) occurring in the setting of major transient risk factors (surgery, trauma or prolonged immobility).”*

3A. A 75-year old woman on warfarin therapy for stroke prevention in the setting of atrial fibrillation requires an elective knee replacement surgery. She has a CHADS2 score of 0. Her INR on admission to the hospital is 2.0. What is the best course of action?

1. Hold warfarin therapy alone
2. Hold warfarin therapy and administer fresh frozen plasma (FFP)
3. Hold warfarin therapy and administer prothrombin complex concentrate (PCC)
4. Hold warfarin therapy and administer both vitamin K and FFP

3B. On a scale of 1-5, with 1 being very low and 5 being very high, what is your level of confidence that your answer to the previous question is in accordance with ASH Choosing Wisely recommendations?

1 2 3 4 5

*Answer: A*

*Based on the recommendation “Don’t administer plasma or prothrombin complex concentrates for non-emergent reversal of vitamin K antagonists (i.e. outside of the setting of major bleeding, intracranial hemorrhage or anticipated emergent surgery).”*

4A. A 46-year old woman develops a large, symptomatic deep venous thrombosis in the right lower extremity after undergoing an elective orthopedic procedure. This is the first time she has had a DVT. She is started on warfarin therapy. What is the most appropriate length of treatment?

1. 6 weeks
2. 3 months
3. 6 months
4. 12 months

4B. On a scale of 1-5, with 1 being very low and 5 being very high, what is your level of confidence that your answer to the previous question is in accordance with ASH Choosing Wisely recommendations?

1 2 3 4 5

*Answer: B*

*Based on the recommendation “Don’t treat with an anticoagulant for more than three months in a patient with a first venous thromboembolism (VTE) occurring in the setting of a major transient risk factor.”*

5A. A 28-year old gentleman with sickle cell disease and baseline hemoglobin of 6.5 presents with an acute pain crisis and a hemoglobin of 5.5. He is hemodynamically stable. In addition to pain management, gentle hydration, and oxygenation, what is the most appropriate course of action?

1. Transfuse crossmatched red cells to a goal hemoglobin of at least 7
2. Transfuse C, E, and Kell matched red cells to a goal hemoglobin of at least 7
3. Do not transfuse red cells at this time
4. Transfuse 2 units of O negative uncrossmatched red cells given the severity of his anemia

5B. On a scale of 1-5, with 1 being very low and 5 being very high, what is your level of confidence that your answer to the previous question is in accordance with ASH Choosing Wisely recommendations?

1 2 3 4 5

*Answer: C*

*Based on the recommendation “Don’t routinely transfuse patients with sickle cell disease (SCD) for chronic anemia or uncomplicated pain crisis without an appropriate clinical indication.”*

6A. A 74-year old gentleman is hospitalized for pneumonia and decompensates requiring intensive care unit transfer, intubation, and mechanical ventilation in addition to broad-spectrum antibiotics. One day after admission, his platelet count is decreased from 250,000/uL (preoperative) to 9,000/uL. There is no evidence of thrombosis. What is the most appropriate course of action?

1. Do not test or treat for heparin-induced thrombocytopenia
2. Send a PF4 test for heparin-induced thrombocytopenia, and start empiric treatment with argatroban or bivalirudin
3. Send a PF4 test for heparin-induced thrombocytopenia, but do not start any treatment unless the result is positive
4. Send a PF4 and a serotonin release assay to test for heparin-induced thrombocytopenia, and only start treatment if both are positive

6B. On a scale of 1-5, with 1 being very low and 5 being very high, what is your level of confidence that your answer to the previous question is in accordance with ASH Choosing Wisely recommendations?

1 2 3 4 5

*Answer: A*

*Based on the recommendation “Don’t test or treat for suspected heparin-induced thrombocytopenia (HIT) in patients with a low pre-test probability of HIT.” – this patient is at LOW risk based on his 4T score (timing of fall is too early for the typical 5-10 day window in HIT, degree of thrombocytopenia is too significant, he has no thromboses, and there are other factors that could cause the thrombocytopenia such as the cardiac bypass surgery)*

7A. A 56-year old woman with known immune thrombocytopenia who has required prednisone in the past for disease control but is currently not on any treatment presents for follow-up; her platelet count has fallen from 80,000/uL at the last visit 3 months prior to 50,000/uL at this current visit. She does not have any bleeding symptoms. What do you recommend in terms of next steps?

1. Start a course of prednisone or dexamethasone
2. Give a 1-time dose of IVIg and start prednisone or dexamethasone
3. Return in 2-4 weeks with a repeat platelet count
4. Recommend either rituximab or splenectomy

7B. On a scale of 1-5, with 1 being very low and 5 being very high, what is your level of confidence that your answer to the previous question is in accordance with ASH Choosing Wisely recommendations?

1 2 3 4 5

*Answer: C*

*Based on the recommendation “Don’t treat patients with immune thrombocytopenic purpura (ITP) in the absence of bleeding or a very low platelet count.” Very low platelet count is generally accepted as 30,000/uL or below.*

8A. A 20-year old woman complains of fatigue and reports severe menorrhagia. Complete blood count demonstrates a hemoglobin of 7.0 g/dL with an MCV of 70, and further testing shows a ferritin level of 2. Her heart rate and blood pressure are normal. What do you recommend?

1. A course of iron therapy started immediately
2. Transfusion of 1 unit of packed red cells and a course of intravenous iron
3. Transfusion of 1 unit of packed red cells followed by oral iron supplementation
4. Consideration of immediate uterine ablation (or hysterectomy if she does not wish children)

8B. On a scale of 1-5, with 1 being very low and 5 being very high, what is your level of confidence that your answer to the previous question is in accordance with ASH Choosing Wisely recommendations?

1. 2 3 4 5

*Answer: A*

*Based on the recommendation “Don't transfuse red blood cells for iron deficiency without hemodynamic instability” from the American Association of Blood Banks*

9A. A 65-year old gentleman with a history of COPD is hospitalized with a pneumonia. He receives appropriate antibiotic treatment and his admission and next-day complete blood count and chemistry testing shows no significant abnormalities. How often should he undergo CBC and electrolyte testing during his hospitalization?

1. Every day
2. Every other day
3. Twice a day
4. If his clinical condition changes

9B. On a scale of 1-5, with 1 being very low and 5 being very high, what is your level of confidence that your answer to the previous question is in accordance with ASH Choosing Wisely recommendations?

1. 2 3 4 5

*Answer: D*

*Based on the recommendation “Don't perform repetitive complete blood count (CBC) and chemistry testing in the face of clinical and lab stability” from the Society for Hospital Medicine/Adult Hospital Medicine*

10A. A 25-year old woman with asthma presents with chest tightness and shortness of breath. She has used her albuterol rescue inhaler 4 times at home without relief of her symptoms, and this is atypical for her. She has a heart rate of 110 and her other vital signs are stable. She has no history of DVT or current signs and symptoms of DVT, no history of malignancy, and reports no hemoptysis. Would you recommend imaging for pulmonary embolism, and why or why not?

1. Yes, because her asthma symptoms did not improve with her rescue inhaler
2. Yes, because she has tachycardia and this can be the only sign of pulmonary embolism
3. No, because an asthma exacerbation is more likely than a pulmonary embolism
4. No, because she is not at moderate-high risk for pulmonary embolism by Wells’ criteria

10B. On a scale of 1-5, with 1 being very low and 5 being very high, what is your level of confidence that your answer to the previous question is in accordance with ASH Choosing Wisely recommendations?

1 2 3 4 5

*Answer: D*

*Based on the recommendation “Don't image for suspected pulmonary embolism (PE) without moderate or high pre-test probability of PE” from the American College of Radiology.*
