# Supplementary material for: Internal medicine trainees' knowledge and confidence in using the American Society of Hematology Choosing Wisely guidelines in hemostasis, thrombosis, and non-malignant hematology
Source: PLoS One. 2018 May 16;13(5):e0197414. doi: 10.1371/journal.pone.0197414 (PMC5955511; doi:10.1371/journal.pone.0197414)
Supplement: S3 File — (DOCX) [file pone.0197414.s003.docx]

4566 Marshall Resident Knowledge

Q1 1A.  A 65-year old patient with a history of leukemia and no history of cardiac disease recently received chemotherapy and presents with a hemoglobin of 6.9.  He is admitted to the hospital for management.  What is your recommended course of action?

- A. Transfuse 2 units of packed red cells (1)
- B. Transfuse 1 unit of packed red cells (2)
- C. Start erythropoietin stimulating agent (ESA) such as Epogen (3)
- D. Give an infusion of intravenous iron (4)

Q2 1B.  On a scale of 1 to 5, with 1 being very low and 5 being very high, what is your level of confidence that your answer to the above question is in accordance with current evidence-based guidelines?

- Very low 1 (1)
- 2 (2)
- 3 (3)
- 4 (4)
- Very high 5 (5)

Q3 2A.  Two days after being hospitalized for a motor vehicle accident, a 45-year old gentleman develops lower extremity swelling and is diagnosed with a deep venous thrombosis.  He does not know of any family history of major venous thromboembolism.  He is started on heparin (as bridging agent) and warfarin therapy.  What is the most appropriate course of action in terms of further workup?

- A. Send factor V Leiden and prothrombin gene mutation testing only (1)
- B. Send protein C and S and antithrombin testing only (2)
- C. Send a complete thrombophilia workup (Factor V Leiden, prothrombin gene mutation, protein C and S levels, antithrombin level, and antiphospholipid antibody testing) (3)
- D. Do not send any thrombophilia testing (4)

Q4 2B.  On a scale of 1 to 5, with 1 being very low and 5 being very high, what is your level of confidence that your answer to the above question is in accordance with current evidence-based guidelines?

- Very low 1 (1)
- 2 (2)
- 3 (3)
- 4 (4)
- Very high 5 (5)

Q5 3A.  A 75-year old woman on warfarin therapy for stroke prevention in the setting of atrial fibrillation requires an elective knee replacement surgery.  She has a CHADS2 score of 0.  Her INR on admission to the hospital is 2.0.  What is the best course of action?

- A. Hold warfarin therapy alone (1)
- B. Hold warfarin therapy and administer fresh frozen plasma (FFP) (2)
- C. Hold warfarin therapy and administer prothrombin complex concentrate (PCC) (3)
- D. Hold warfarin therapy and administer both vitamin K and FFP (4)

Q6 3B.  On a scale of 1 to 5, with 1 being very low and 5 being very high, what is your level of confidence that your answer to the above question is in accordance with current evidence-based guidelines?

- Very low 1 (1)
- 2 (2)
- 3 (3)
- 4 (4)
- Very high 5 (5)

Q7 4A.  A 46-year old woman develops a large, symptomatic deep venous thrombosis in the right lower extremity after undergoing an elective orthopedic procedure.  This is the first time she has had a DVT.  She is started on warfarin therapy.  What is the most appropriate length of treatment?

- A. 6 weeks (1)
- B. 3 months (2)
- C. 6 months (3)
- D. 12 months (4)

Q8 4B.  On a scale of 1 to 5, with 1 being very low and 5 being very high, what is your level of confidence that your answer to the above question is in accordance with current evidence-based guidelines?

- Very low 1 (1)
- 2 (2)
- 3 (3)
- 4 (4)
- Very high 5 (5)

Q9 5A.  A 28-year old gentleman with sickle cell disease and baseline hemoglobin of 6.5 presents with an acute pain crisis and a hemoglobin of 5.5.  He is hemodynamically stable.  In addition to pain management, gentle hydration, and oxygenation, what is the most appropriate course of action?

- A. Transfuse crossmatched red cells to a goal hemoglobin of at least 7 (1)
- B. Transfuse C, E, and Kell matched red cells to a goal hemoglobin of at least 7 (2)
- C. Do not transfuse red cells at this time (3)
- D. Transfuse 2 units of O negative uncrossmatched red cells given the severity of his anemia (4)

Q10 5B.  On a scale of 1 to 5, with 1 being very low and 5 being very high, what is your level of confidence that your answer to the above question is in accordance with current evidence-based guidelines?

- Very low 1 (1)
- 2 (2)
- 3 (3)
- 4 (4)
- Very high 5 (5)

Q11 6A.  A 74-year old gentleman is hospitalized for pneumonia and decompensates requiring intensive care unit transfer, intubation, and mechanical ventilation in addition to broad-spectrum antibiotics.  One day after admission, his platelet count is decreased from 250,000/uL (preoperative) to 9,000 uL.  There is no evidence of thrombosis.  What is the most appropriate course of action?

- A. Do not test or treat for heparin-induced thrombocytopenia (1)
- B. Send a PF4 test for heparin-induced thrombocytopenia, and start empiric treatment with argatroban or bivalirudin (2)
- C. Send a PF4 test for heparin-induced thrombocytopenia, but do not start any treatment unless the result is positive (3)
- D. Send a PF4 and a serotonin release assay to test for heparin-induced thrombocytopenia, and only start treatment if both are positive (4)

Q12 6B.  On a scale of 1 to 5, with 1 being very low and 5 being very high, what is your level of confidence that your answer to the above question is in accordance with current evidence-based guidelines?

- Very low 1 (1)
- 2 (2)
- 3 (3)
- 4 (4)
- Very high 5 (5)

Q13 7A.  A 56-year old woman with known immune thrombocytopenia who has required prednisone in the past for disease control but is currently not on any treatment presents for follow-up; her platelet count has fallen from 80,000/uL at the last visit 3 months prior to 50,000/uL at this current visit.  She does not have any bleeding symptoms.  What do you recommend in terms of next steps?

- A. Start a course of prednisone or dexamethasone (1)
- B. Give a 1-time dose of IVIg and start prednisone or dexamethasone (2)
- C. Return in 2 to 4 weeks with a repeat platelet count (3)
- D. Recommend either rituximab or spenectomy (4)

Q14 7B.  On a scale of 1 to 5, with 1 being very low and 5 being very high, what is your level of confidence that your answer to the above question is in accordance with current evidence-based guidelines?

- Very low 1 (1)
- 2 (2)
- 3 (3)
- 4 (4)
- Very high 5 (5)

Q15 8A.  A 20-year old woman complains of fatigue and reports severe menorrhagia.  Complete blood count demonstrates a hemoglobin of 7.0 g/dL with an MCV of 70, and further testing shows a ferritin level of 2.  Her heart rate and blood pressure are normal.  What do you recommend?

- A. A course of iron therapy started immediately (1)
- B. Transfusion of 1 unit of packed red cells and a course of intravenous iron (2)
- C. Transfusion of 1 unit of packed red cells followed by oral iron supplementation (3)
- D. Consideration of immediate uterine ablation (or hysterectomy if she does not wish children) (4)

Q16 8B.  On a scale of 1 to 5, with 1 being very low and 5 being very high, what is your level of confidence that your answer to the above question is in accordance with current evidence-based guidelines?

- Very low 1 (1)
- 2 (2)
- 3 (3)
- 4 (4)
- Very high 5 (5)

Q17 9A.  A 65-year old gentleman with a history of COPD is hospitalized with a pneumonia.  He receives appropriate antibiotic treatment and his admission and next-day complete blood count and chemistry testing shows no significant abnormalities.  How often should he undergo CBC and electrolyte testing during his hospitalization?

- A. Every day (1)
- B. Every other day (2)
- C. Twice a day (3)
- D. If his clinical condition changes (4)

Q18 9B.  On a scale of 1 to 5, with 1 being very low and 5 being very high, what is your level of confidence that your answer to the above question is in accordance with current evidence-based guidelines?

- Very low 1 (1)
- 2 (2)
- 3 (3)
- 4 (4)
- Very high 5 (5)

Q19 10A.  A 25-year old woman with asthma presents with chest tightness and shortness of breath.  She has used her albuterol rescue inhaler 4 times at home without relief of her symptoms, and this is atypical for her.  She has a heart rate of 110 and her other vital signs are stable.  She has no history of DVT or current signs and symptoms of DVT, no history of malignancy, and reports no hemoptysis.  Would you recommend imaging for pulmonary embolism, and why or why not?

- A. Yes, because her asthma symptoms did not improve with her rescue inhaler (1)
- B. Yes, because she has tachycardia and this can be the only sign of pulmonary embolism (2)
- C. No, because an asthma exacerbation is more likely than a pulmonary embolism (3)
- D. No, because she is not at moderate-high risk for pulmonary embolism by Wells' criteria (4)

Q20 10B.  On a scale of 1 to 5, with 1 being very low and 5 being very high, what is your level of confidence that your answer to the above question is in accordance with current evidence-based guidelines?

- Very low 1 (1)
- 2 (2)
- 3 (3)
- 4 (4)
- Very high 5 (5)

Q21 BACKGROUND

Q22 Sex:

- Male (1)
- Female (2)

Q23 Residency Site:

- Bringham and Women's (1)
- Massachusetts General Hospital (2)
- Mayo Clinic Rochester (3)
- University of Michigan (4)
- Yale (5)

Q24 Prelim or Categorical?

- Prelim (1)
- Categorical (2)
- Other, please specify: (3) ____________________

Q25 Year in residency:

- PGY1 (1)
- PGY2 (2)
- PGY3 (3)
- Other, please specify: (4) ____________________

Q26 Have you done a hematology rotation yet?

- Yes (1)
- No (2)

Display This Question:

If Have you done a hematology rotation yet? Yes Is Selected

Q27 If so, which ones?  (Mark all that apply.)

- Hematology Consult (1)
- Outpatient hematology (non-malignant) (2)
- Outpatient hematology (malignant) (3)
- Inpatient hematology (malignant) (4)
- Other, please specify: (5) ____________________

Q28 Are you aware that the American Society of Hematology (ASH) has published a set of "Choosing Wisely" guidelines in an attempt to reduce waste and cost in health care?

- Yes (1)
- No (2)

Q29 What is your intended specialty?

- Hematology/oncology (1)
- Specialty other than hematology/oncology (2)
- Unknown (3)

Q30 Thank you for your time!   Please click SUBMIT to record your responses.
